# Gray Matter Volume Loss in Parkinson's Disease Psychosis and Cannabinoid Receptor Gene Expression in the Brain

**DOI:** 10.1002/mds.70222

**Published:** 2026-02-20

**Authors:** Sara Pisani, František Váša, Latha Velayudhan, Sagnik Bhattacharyya

**Affiliations:** ^1^ Department of Psychosis Studies Institute of Psychiatry, Psychology & Neuroscience, King's College London London United Kingdom; ^2^ Department of Neuroimaging Institute of Psychiatry, Psychology & Neuroscience, King's College London London United Kingdom; ^3^ Department of Psychological Medicine, Centre for Healthy Brain Ageing Institute of Psychiatry, Psychology & Neuroscience, King's College London London United Kingdom

**Keywords:** endocannabinoid receptors, meta‐regression, Parkinson's disease, psychosis

## Abstract

**Background:**

Serotonergic and dopaminergic pathways are implicated in Parkinson's disease psychosis (PDP), but preliminary evidence also implicates the endocannabinoid system (ECS).

**Objectives:**

We examined the association of gray matter volume loss in PDP patients with brain expression of the genes coding for CB1 (cannabinoid type 1) and CB2 cannabinoid receptors obtained from an independent sample.

**Methods:**

Hedges' *g* effect sizes indexing gray matter volume loss were extracted from a previous meta‐analytic comparison of PDP and PD without psychosis. The relationship of cortical and subcortical CB1 and CB2 gene expression in 6 healthy individuals extracted from the Allen Human Brain Atlas with meta‐analytic estimates of PDP‐related volume loss was examined correcting for spatial autocorrelation.

**Results:**

There was a significant association between gray matter volume loss in PDP and CB1 (*r* = 0.337, *t*(76) = 3.122, *P* < 0.001) but not CB2 (*P* = 0.132, corrected) receptor brain expression.

**Conclusions:**

This finding underscores the importance of investigating the role of the ECS in PD. © 2026 The Author(s). *Movement Disorders* published by Wiley Periodicals LLC on behalf of International Parkinson and Movement Disorder Society.

Hallucinations and delusions are distressing and burdensome nonmotor symptoms in Parkinson's Disease (PD), collectively referred to as PD psychosis (PDP). They present an unmet clinical need and are associated with increased mortality, poor quality of life, and dementia.^1^, ^2^, ^3^, ^4^ Dopaminergic,^5^, ^6^ serotonergic (eg, serotoninergic availability of 5‐hydroxytryptamine receptor 1A and 5‐hydroxytryptamine receptor 2A),^7^, ^8^, ^9^ and cholinergic^10^ pathways have been implicated in PDP. Recently, there is growing interest in the endocannabinoid system (ECS), a lipid signaling system, in this regard with emerging evidence of alterations in its components in people with PD and in preclinical models of the disorder.^11^, ^12^, ^13^, ^14^ In addition, there is evidence of the antipsychotic potential of cannabidiol, a functional antagonist of cannabinoid type 1 (CB1) receptors, in PDP^15^ and in people in various stages of psychosis.^16^, ^17^, ^18^ Although this is consistent with independent evidence of involvement of the ECS in psychotic disorders,^19^, ^20^ the role of the ECS in PDP remains unclear. Therefore, with this work, we aimed to investigate whether brain expression of the key endocannabinoid receptors (ie, CB1 and CB2) may be associated with gray matter volume alterations in PDP patients. To achieve this, we carried out secondary analysis using data from prior meta‐analytic work where we compared gray matter volume between PD patients with and without psychosis.^7^ Here, we explored the association of gray matter volume loss in PD psychosis with the central expression of the genes coding for CB1 and CB2 receptors obtained from an independent sample derived from the Allen Human Brain Atlas,^21^ which includes a microarray expression data in tissue samples from 6 healthy brain donors.

## Patients and Methods

### Study Eligibility

Data for this work were derived from our previous coordinate‐based meta‐analysis, and a detailed methodology has been described before. In brief, after systematic database search (search date: June 7, 2021), structural magnetic resonance imaging (MRI) studies providing data (ie, peak coordinates or statistical maps in standardized space, eg, Talairach or Montreal Neurological Institute) from comparisons of gray matter volume in PDP and PD patients without psychosis (PDnP) using a voxel‐based morphometry approach were included. The original search led to 13,067 results (including 13 results from Neurosynth search and citation search). After removal of duplicates (3746) and ineligible studies (9311), the final pool consisted of 10 MRI studies.

### Data Synthesis and Analysis

We examined the association between the spatial pattern of PDP‐related gray matter volume loss as indexed by Hedges' *g* effect sizes (from both unadjusted and levodopa equivalent daily dose [LEDD] adjusted meta‐analyses), computed from peak coordinate data extracted from eligible MRI studies using Seed‐Based *d* Mapping with Permutation of Subject Images (*SDM‐PSI*, version *6.21*),^22^ with the messenger RNA (mRNA) microarray gene expression of CB1 and CB2 receptor genes extracted and processed as before,^21^, ^23^, ^24^ using Pearson's correlation coefficient across cortical and subcortical regions (see Supplementary Methods for details in Data S1). To estimate the statistical significance of these relationships, we used a null model framework that accounts for the similarity in values between proximal brain regions, due to spatial autocorrelation.^25^ Analyses were carried out using the open‐source *Python*‐based platform “Brain Surrogate Maps with Autocorrelated Spatial Heterogeneity” (ie, BrainSMASH).^26^, ^27^ For each pairwise correlation between brain maps, we compared the empirical correlation coefficient to a null distribution generated by correlating one (empirical) map to 10,000 surrogate versions of the second map, which randomize the spatial pattern of values while preserving spatial autocorrelation across both cortical and subcortical regions. The null distribution was used to obtain a *P*‐value, defined as the proportion of surrogate correlation coefficients that are greater than or equal to the empirical correlation.

## Results

### Meta‐Analysis Results

Ten structural MRI studies were included (references are available in the Supplementary Results in Data S1), reporting on 211 PDP patients (age, mean ± standard deviation [SD] = 69.01 ± 4.90; 52.1% male, LEDD, mean ± SD = 651.94 ± 318.14, motor symptom severity [Movement Disorder Society‐Sponsored Revision of the Unified Parkinson's Disease Rating Scale, MDS‐UPDRS, Part III], mean ± SD = 28.72 ± 11.69) and 298 PDnP patients (age, mean ± SD = 67.34 ± 4.59; 49.1% male, LEDD, mean ± SD = 577.06 ± 263.11, motor symptom severity [MDS‐UPDRS, Part III], mean ± SD = 25.94 ± 8.63). Detailed meta‐analysis results are reported in our prior publication.^7^ Overall, patients with PDP had lower gray matter volume in parietal, occipital, and temporal areas compared to PDnP. The results remained unchanged after controlling for PD medications (expressed as LEDD) and cognitive status (see Supplementary Results from more details in Data S1).

### Meta‐Regression

Separate pairwise correlations (uncorrected for spatial autocorrelation) revealed a significant association between whole‐brain gray matter volume loss unadjusted for PD medications (LEDD) and local expression of the CB1 receptor (*r* = 0.337, *t*(76) = 3.122, *P* = 0.003), and a trend toward association with the CB2 receptor (*r* = 0.221, *t*(76) = 1.976, *P* = 0.052). When we entered the LEDD‐adjusted Hedges' *g* effect sizes, CB1 results remained significant (*r* = 0.357, *t*(76) = 3.328, *P* = 0.001), and there was a significant association between CB2 and gray matter volume loss (*r* = 0.229, *t*(76) = 2.055, *P* = 0.043).

**FIG. 1:**
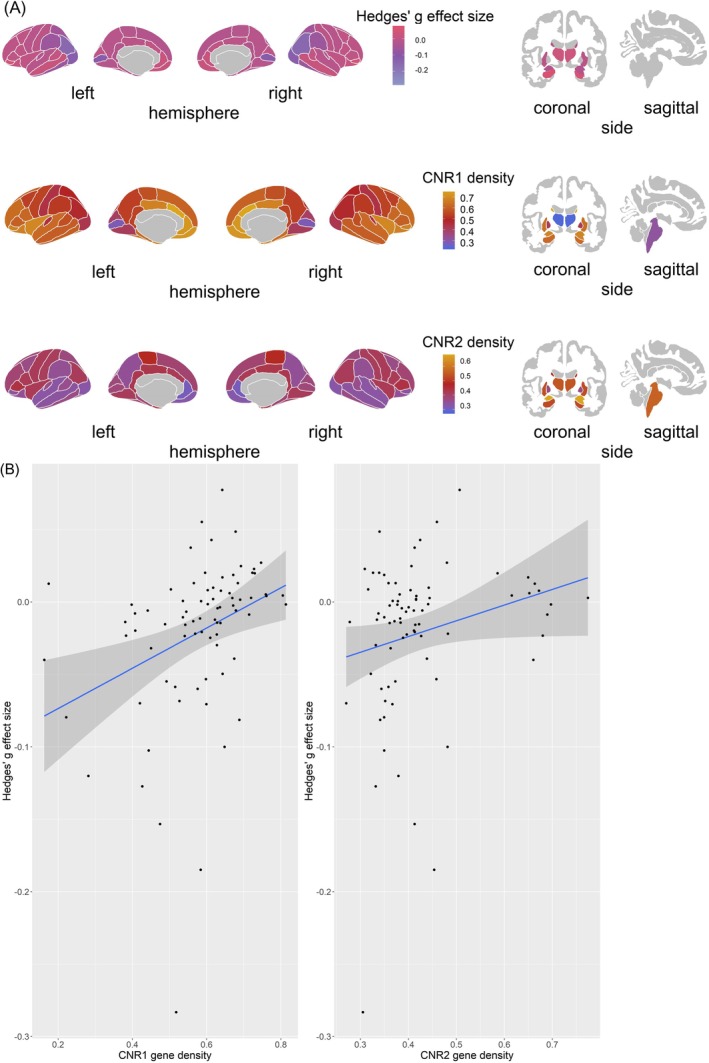
(**A**) Hedges' *g* effect size and gene expression density of CB1 (CNR1) and CB2 (CNR2) receptors in cortical and subcortical areas derived, parcellated across the Desikan–Killiany atlas and subcortical regions. (**B**) Scatterplots showing the relationship between CB1 and CB2 receptors and Hedges' *g* effect size, adjusted for LEDD (levodopa equivalent daily dose). [Color figure can be viewed at wileyonlinelibrary.com]

Correction for spatial autocorrelation (using BrainSMASH) associated with the pairwise Pearson's correlation coefficients showed consistent results for the correlation of CB1 gene expression with unadjusted and LEDD‐adjusted effect sizes (unadjusted, *P* < 0.001; LEDD adjusted, *P* = 0.015). No association between cortical volume loss and CB2 was observed after correcting for spatial autocorrelation (unadjusted, *P* = 0.090; LEDD adjusted, *P* = 0.132) (Fig. 1).

## Discussion

Here, using summary effect‐size data on group difference in brain volume from our previous^7^ meta‐analytic comparison of gray matter volume alterations in PDnP, we examined the association between the brain expression of the genes coding for the main cannabinoid receptors (extracted from the Allen Human Brain Atlas) and meta‐analytic estimate of effect size of cortical volume difference in PDP (relative PDnP). Our results show a positive association between CB1 gene expression density and regional cortical volume change in PDP compared to those without psychosis. Our meta‐analysis showed lower cortical volumes across several brain regions in PDP compared to PDnP.^7^ Results of the present analyses suggest that the greater the regional CB1 gene expression density, the greater the effect size of gray matter cortical and subcortical volume change in PDP relative to PDnP. This remained significant even after controlling for LEDD and for spatial autocorrelation. However, no association was found with CB2 gene expression density. This may suggest that the extent of cortical and subcortical gray matter volume loss that has been reported in PD patients who develop psychosis compared to those who do not^8^, ^28^ and that was also evident from our meta‐analytic examination^7^ may be linked to local expression of the CB1 receptor, consistent with positron emission tomography (PET) evidence of lower subcortical CB1 availability in people with PD.^14^ Positive relationship, as observed here, may merely reflect brain volume loss because of the neurodegenerative process resulting in both lower gray matter volume and reduced CB1 density in PDP. Presynaptic CB1 receptors modulate the release of different neurotransmitters, including glutamate.^29^ Therefore, loss of presynaptic CB1 receptors may also contribute to greater gray matter loss in PDP as a result of poor homeostatic control of excitotoxic neurotransmission. However, both these possibilities remain to be tested. Of note, greater severity of hallucinations and delusions in psychosis (unrelated to PD) has been associated with reduced CB1 availability using PET.^30^

Understanding the role of ECS in PDP could provide new insights into its pathophysiology and potential therapeutic targets. We did not find a significant relationship between gray matter volume and CB2 gene expression density, which could be due to the lower availability of expression of CB2 receptors in the brain compared to CB1 receptors. Both CB1 and CB2 receptors are g‐protein‐coupled receptors; however, the latter is mostly expressed in peripheral organs involved in immune functions, as well as microglia and astrocytes in people with Alzheimer's disease and multiple sclerosis.^31^, ^32^, ^33^ Conversely, CB1 receptors are abundantly expressed in the brain, specifically in the basal ganglia, limbic regions (eg, amygdala), hippocampus, cerebellum, and widely across the cortex.^34^, ^35^ Because CB1 receptors are more widely expressed in the cortex compared to CB2 receptors, it is possible that CB1 receptors may be involved in the pathophysiology of these symptoms in PD. It is worth noting that reduced CB1 receptor levels have been reported in people with psychotic disorders such as schizophrenia,^30^, ^36^ consistent with experimental evidence of acute induction of psychotic symptoms by delta‐9‐tetrahydrocannabinol, a partial agonist of CB1 receptor.^37^, ^38^ Whether alterations in components of the ECS such as CB1 receptors may also play a role in the pathophysiology of psychotic symptoms in PD remains unclear. Future research should therefore examine whether the ECS and its components are differentially altered in people with PD with psychosis compared to those without psychosis using approaches such as PET imaging to obtain an estimate of the availability of CB1 receptors in vivo.

This study has some limitations. First, the results reported here are drawn from secondary data analysis: we correlated measure from a pooled data synthesis approach (ie, a coordinate‐based meta‐analysis) in different samples of PD patients with and without psychosis with a proxy measure for CB1 density derived from 6 healthy donors (ie, mRNA expression from the Allen Human Brain Atlas^21^, ^23^). As the brain volume data and the CB1 expression data were not obtained from the same individuals, caution is warranted as we did not examine the relationship between brain volume alteration and CB1 expression in the same individuals. Nevertheless, previous studies have applied a similar approach and have shown consistency of the spatial architecture of receptor systems in the brain across individuals.^39^, ^40^ In addition, as the gray matter volume estimates were derived from a coordinate‐based meta‐analysis, we relied on available peak coordinates that represent a less effective approach.^41^ Nevertheless, in this secondary analysis, our key results remained significant even after we controlled for additional variability due to spatial autocorrelation and show a relationship between the spatial distribution of the effect size of gray matter volume change in people with PD psychosis and the spatial pattern of average brain regional CNR1 gene expression levels, a proxy measure of CB1 density, from an independent dataset of postmortem brains of healthy individuals.

## Author Roles

(1) Research Project: A. Conception, B. Organization, C. Execution; (2) Statistical Analysis: A. Design, B. Execution, C. Review and Critique; (3) Manuscript Preparation: A. Writing of the First Draft, B. Review and Critique.

S.P.: 1A, 1B, 1C, 2A, 2B, 2C, 3A, 3B.

F.V.: 2A, 2B, 2C, 3A, 3B.

L.V.: 1A, 2C, 3A, 3B.

S.B.: 1A, 1B, 1C, 2A, 2B, 2C, 3A, 3B.

## Full financial disclosures of all authors for the past 12 months

S.B. is supported by grants from the National Institute of Health Research (NIHR) Efficacy and Mechanism Evaluation scheme and Parkinson's UK. S.B. has participated in advisory boards for or received honoraria as a speaker from Reckitt Benckiser, EmpowerPharm/SanteCannabis, and Britannia Pharmaceuticals. All these honoraria were received as contributions toward research support through King's College London and not personally. S.B. has also collaborated with Beckley Canopy Therapeutics/Canopy Growth (investigator‐initiated research), which provided the study drug free of charge (Parkinson's UK) and NIHR (BRC) funded research. The views expressed are those of the authors and not necessarily those of the NHS, the NIHR, or the Department of Health. L.V. has collaborated with Beckley Canopy Therapeutics/Canopy Growth (investigator‐initiated research), which provided the study drug free of charge (Parkinson's UK) and NIHR (BRC) funded research. F.V. is funded by the Bill and Melinda Gates Foundation UNITY project (INV‐032788), the MRC (MR/Z503812/1), and the NIHR Maudsley Biomedical Research Centre. None of these funding sources directly supported F.V.'s contributions to this work. S.B. and L.V. are in receipt of a grant from Parkinson’ UK (I‐1901). S.P. PhD studentship was supported by Parkinson's UK.

## Financial Disclosures and Conflicts of Interest

Author disclosures are available in the Supporting Information.

## Supporting information


**Data S1.** Supporting Information.

## Data Availability

The summary effect‐size data used in this work are derived from a previously published systematic review and meta‐analysis^7^; therefore, they are available upon request to the corresponding author. The CB1 and CB2 receptor gene expression data used in this study are also accessible to all researchers from the Allen Human Brain Atlas website (https://human.brain-map.org/).
