# Supplementary material for: Gray Matter Volume Loss in Parkinson's Disease Psychosis and Cannabinoid Receptor Gene Expression in the Brain
Source: Mov Disord. 2026 Feb 20;41(6):1566–71. doi: 10.1002/mds.70222 (PMC13307235; doi:10.1002/mds.70222)
Supplement: Supplementary file 1 — Data S1. Supporting Information. [file MDS-41-1566-s001.docx]

**Supplementary Materials**

**Grey matter volume loss in Parkinson’s disease Psychosis and cannabinoid receptor gene expression in the brain**

Sara Pisani, František Váša, Latha Velayudhan, Sagnik Bhattacharyya

Table of Contents

[**Supplementary Methods** 2](#_Toc217117926)

[Study eligibility 2](#_Toc217117927)

[Data synthesis and analysis 2](#_Toc217117928)

[**Supplementary Results** 3](#_Toc217117929)

[Meta analysis results 3](#_Toc217117930)

[**Discussion** 4](#_Toc217117931)

[**Supplementary Table** 4](#_Toc217117932)

[**References** 6](#_Toc217117933)

# **Supplementary Methods**

## Study eligibility

Data for this work were derived from our previous coordinate-based meta-analysis and detailed methodology has been described before^1^. After a systematic database search on PubMed, Web of Science and Emabase (search date 7^th^ June 2021), structural magnetic resonance imaging (MRI) studies providing data (i.e., peak coordinates or statistical maps in standardised space e.g., Talariach or MNI) from comparisons of grey matter volume in PD patients with psychosis (PDP) and without psychosis (PDnP) using a voxel-based morphometry (VBM) approach were included.

The original search led to 13,067 results (including 13 results from Neurosynth search and citation search). After removal of duplicates (3,746) and ineligible studies (9,311), the final pool was 10 MRI studies.

## Data synthesis and analysis

As reported before^1^, we first conducted a coordinate-based meta-analysis using peak coordinate data extracted manually and/or statistical maps (supplied by authors) from eligible MRI studies using Seed-based *d* Mapping with Permutation of Subject Images (SDM-PSI, v6.21) ^2^ and a random-effects approach. Data used in the original meta-analysis included peak coordinates extracted manually from nine studies ^3-11^ and T maps provided by authors of one study^12^. For the present study, we extracted the previously estimated Hedges’ *g* effect-sizes from the centroid of the SDM-PSI meta-analytic map (reported previously^1^) for all cortical and subcortical regions following a parcellation scheme implemented in FreeSurfer (https://surfer.nmr.mgh.harvard.edu/fswiki/FreeSurferWiki). We also extracted CB1 and CB2 receptor gene expression data from the Allen Human Brain Atlas which includes microarray expression data in tissue samples from six healthy adult human brains with more than 20,000 genes quantified across cortical and subcortical regions, including brainstem and cerebellum extracted and processed (probe-to-gene re-annotation, sample-to-region matching and extraction) as before ^13^ ^14,15^. The brain parcellation used included the original 68 cortical regions in the Desikan Killiany atlas ^16^ and 15 subcortical regions as implemented in the FreeSurfer *aseg* atlas^17^. Gene expression data were not available for the right frontal pole and the right temporal pole, and volume data from the SDM-PSI analysis was unavailable for the bilateral pallidum and the brainstem, therefore these regions were excluded from the analysis. Hence, subsequent analyses focused on all 78 cortical and subcortical regions for which data was available. We examined the association between the spatial pattern of PDP-related grey matter volume loss as indexed by the extracted Hedges’ *g* effect-sizes from the centroid of the SDM-PSI meta-analytic map ^1^ parcellated across cortical and subcortical brain regions ^16,17^ with the mRNA microarray gene expression of CB1 and CB2 receptor genes. We computed the Pearson correlation coefficient across cortical and subcortical regions using Hedges’ *g* effect-sizes extracted from both unadjusted and Levodopa equivalent daily dose (LEDD) adjusted meta-analyses.

# **Supplementary Results**

Ten studies were included in the analysis; nine provided peak coordinates ^3-11^ and one provided T maps^12^. Full results from the meta-analysis on 10 MRI studies are reported in our previous publication^1^. Peak coordinates from these analyses (which have been previously reported^1^) are included as supplementary table below for completeness.

## Meta analysis results

Detailed meta-analysis results have been reported in our prior publication^1^ and are included here for completeness. In summary, compared to PDnP group, patients with PDP had lower grey matter volume in parieto-temporo-occipital regions, namely the right precuneus (voxel number = 1059, Z = −2.990, p = 0.001), bilateral inferior parietal gyri (left, voxel number = 296, Z = −2.449, p = 0.007; right, voxel number = 277, Z = −2.225, p = 0.013), left inferior occipital (voxel number = 262, Z = −2.627, p = 0.004), and right middle temporal (voxel number = 225, Z = −2.664, p = 0.003) gyri. On adjusting for LEDD, the peak showing lower grey matter volume in the right precuneus (voxel number = 1084, Z = −2.952, p = 0.001) remained significant, and there was significant overlap with the peaks observed in the unadjusted analysis (i.e., left angular gyrus extending to left inferior parietal gyrus, voxel number = 389, Z = −2.596, p = 0.004; right inferior temporal gyrus extending to right middle temporal gyrus, voxel number = 353, Z = −0.2921, p = 0.001; the left middle occipital gyrus extending to inferior occipital gyrus, voxel number = 315, Z = −2.902, p = 0.001; the right inferior parietal gyrus, voxel number = 304, Z = −2.525, p = 0.005). None of these regions from either the unadjusted or adjusted analyses remained significant after familywise error correction for multiple testing. Peak coordinates from these analyses (which have been previously reported^1^) are also included as supplementary table. There were no statistically significant peaks in the opposite direction (PDP > PDnP).

# **Discussion**

As the primary focus of the present study is the relationship between the spatial pattern of group difference in brain volume and gene expression, we do not discuss the results of the grey matter volume differences from the original meta-analysis, which have been reported and discussed before^1^. Instead, we focus on the association between gene expression and pooled estimates of grey matter volume difference.

As we correlated the spatial pattern of grey matter volume change across the brain with the spatial pattern of gene expression, it not possible to infer about what this may mean in terms of specific brain regions. Nevertheless, our results suggest that the regions with the greatest volume loss, such as the parieto-temporo-occipital regions may also have the greatest CB1 receptor -related dysfunction. As we have discussed before, the brain regions with lower grey matter volume in PDP map onto brain substrates involved in higher order visual processing and more broadly information processing and are part of the default mode network^1^. Therefore, these results are consistent with some of the hypothesized mechanisms of hallucinations in PDP^18,19^ and the likely role of CB1 receptors^20,21^ in modulating these mechanisms. However, unravelling the precise nature and potential role of cannabinoid pathway dysfunction and confirming its regional distribution in the brain needs PET imaging of CB1 receptor distribution in people with PD with and without psychosis.

# **Supplementary Table**

Meta-analytic peak coordinates showing smaller grey matter volume in Parkinson’s disease psychosis (PDP) patients compared to those without psychosis (PDnP)- unadjusted results as well as results adjusted for Levodopa equivalent daily dose (LEDD).

|  | Peak regions | MNI coordinates | | | Number of voxels | Z score | p value | *I²* | Egger’s test *p* value |
| --- | --- | --- | --- | --- | --- | --- | --- | --- | --- |
|  |  | **x** | **y** | **z** |  |  |  |  |  |
| PDP < PDnP (unadjusted) | |  |  |  |  |  |  |  |  |
|  | Right precuneus (extending to left precuneus, bilateral cuneus) | 10 | -66 | 42 | 1059 | -2.990 | 0.001 | 1.729% | n.s. |
|  | Left inferior parietal gyrus (extending to the left angular gyrus, and supramarginal gyrus) | -48 | -52 | 40 | 296 | -2.449 | 0.007 | 0.849% | n.s |
|  | Right inferior parietal gyrus (extending to right angular gyrus) | 46 | -56 | 44 | 277 | -2.225 | 0.013 | 27.631% | n.s |
|  | Left inferior occipital gyrus | -38 | -84 | -8 | 262 | -2.627 | 0.004 | 11.411% | n.s |
|  | Right middle temporal gyrus (extending to right inferior temporal gyrus) | 52 | -2 | -30 | 225 | -2.664 | 0.003 | 9.657% | n.s |
|  | Right cerebellum, hemispheric lobule VIII (extending to lobule IX) | 18 | -52 | -54 | 92 | -1.983 | 0.023 | 16.551% | n.s |
|  | Left median cingulate / paracingulate gyrus (extending to the right median cingulate and paracingulate gyrus) | -4 | -8 | 46 | 57 | -2.032 | 0.021 | 5.906% | n.s |
|  | Left inferior temporal gyrus | -46 | -8 | -32 | 42 | -1.989 | 0.023 | 0.517% | n.s |
|  | Right supramarginal gyrus | 58 | -38 | 30 | 32 | -2.179 | 0.014 | 8.127% | n.s. |
|  | Right lingual gyrus (extending to the calcarine fissure) | 10 | -54 | 8 | 29 | -2.186 | 0.014 | 1.229% | n.s |
|  | Left postcentral gyrus (extending to left inferior parietal gyrus) | -40 | -36 | 44 | 22 | -2.163 | 0.015 | 5.246% | n.s |
| PDP < PDnP (adjusted for LEDD) | | | | | | | | | |
|  | Right precuneus (extending to the left precuneus and bilateral cuneus) | 6 | -62 | 46 | 1084 | -2.952 | 0.001 | 5.656% | n.s. |
|  | Left angular gyrus (extending to left inferior parietal gyrus) | -50 | -56 | 34 | 389 | -2.596 | 0.004 | 2.443% | n.s. |
|  | Right inferior temporal gyrus (extending to the right middle temporal gyrus and right fusiform) | 50 | -10 | -30 | 353 | -2.921 | 0.001 | 3.859% | n.s. |
|  | Left middle occipital gyrus (extending to the inferior occipital gyrus) | -34 | -84 | 6 | 315 | -2.902 | 0.001 | 1.279% | n.s. |
|  | Right inferior parietal gyrus (extending to the right angular gyrus) | 40 | -56 | 48 | 304 | -2.525 | 0.005 | 3.747% | n.s |
|  | Left median cingulate/paracingulate gyrus (extending to the right median cingulate/paracingulate gyrus and left supplementary motor area) | 2 | 12 | 41 | 144 | -2.206 | 0.013 | 2.152 | n.s |
|  | Right supramarginal gyrus | 56 | -40 | 28 | 117 | -.2693 | 0.003 | 3.343% | n.s. |
|  | Right lingual gyrus (extending to cerebellum, vermis lobule IV/V) | 10 | -52 | 6 | 105 | -2.345 | 0.009 | 1.418% | n.s |
|  | Right middle occipital gyrus | 44 | -76 | 8 | 104 | -2.203 | 0.013 | 0.968% | n.s |
|  | Left inferior temporal gyrus | -46 | -10 | -26 | 95 | -2.270 | 0.011 | 0.526% | n.s |
|  | Right fusiform gyrus (extending to the right cerebellum, hemispheric lobule IV/V) | 24 | -36 | -22 | 85 | -2.275 | 0.011 | 8.312 | n.s |
|  | Left temporal pole | -44 | 8 | -40 | 47 | -2.340 | 0.009 | 0.196% | n.s |
|  | Left inferior parietal gyrus (extending to the left postcentral gyrus) | -38 | -38 | 44 | 39 | -2.538 | 0.005 | 7.669% | n.s |
|  | Left cerebellum hemispheric lobule VI (extending to the left lingual gyrus) | -10 | -78 | -16 | 39 | -2.197 | 0.014 | 3.506% | n.s |
|  | Left supplementary motor area (extending to the left median cingulate/paracingulate gyrus) | -4 | -12 | 48 | 26 | -2.233 | 0.012 | 2.144% | n.s |
|  | Right calcarine fissure (extending to the right lingual gyrus) | 10 | -86 | -2 | 21 | -2.082 | 0.019 | 0.047% | n.s |

*p* value for the coordinates represent uncorrected p value; *I*² indicates the magnitude of between-study heterogeneity as a proportion of total variability within each peak; Publication bias is expressed as Egger’s test *p* value; n.s.: non-significant.

# **References**

1. Pisani S, Gunasekera B, Lu Y, et al. Grey matter volume loss in Parkinson’s disease psychosis and its relationship with serotonergic gene expression: A meta-analysis. *Neuroscience & Biobehavioral Reviews* 2023; **147**: 105081.

2. Albajes-Eizagirre A, Solanes A, Vieta E, Radua J. Voxel-based meta-analysis via permutation of subject images (PSI): theory and implementation for SDM. *Neuroimage* 2019; **186**: 174-84.

3. Bejr-Kasem H, Pagonabarraga J, Martinez-Horta S, et al. Disruption of the default mode network and its intrinsic functional connectivity underlies minor hallucinations in Parkinson's disease. *Movement Disorders* 2019; **34**(1): 78-86.

4. Bejr-Kasem H, Sampedro F, Marín-Lahoz J, Martínez-Horta S, Pagonabarraga J, Kulisevsky J. Minor hallucinations reflect early gray matter loss and predict subjective cognitive decline in parkinson's disease. *Eur J Neurol* 2020.

5. Firbank MJ, Parikh J, Murphy N, et al. Reduced occipital GABA in Parkinson disease with visual hallucinations. *Neurology* 2018; **91**(7): e675.

6. Goldman JG, Stebbins GT, Dinh V, et al. Visuoperceptive region atrophy independent of cognitive status in patients with Parkinson’s disease with hallucinations. *Brain* 2014; **137**(3): 849-59.

7. Lawn T. Cerebellar correlates of visual hallucinations in Parkinson's disease and Charles Bonnet Syndrome. *Cortex* 2021; **135**: 311-25.

8. Pagonabarraga J, Soriano-Mas C, Llebaria G, López-Solà M, Pujol J, Kulisevsky J. Neural correlates of minor hallucinations in non-demented patients with Parkinson's disease. *Parkinsonism & related disorders* 2014; **20**(3): 290-6.

9. Ramirez-Ruiz B, Junque C, Marti MJ, Valldeoriola F, Tolosa E. Cognitive changes in Parkinson's disease patients with visual hallucinations. *Dement Geriatr Cogn Disord* 2007; **23**(5): 281-8.

10. Shin S, Lee JE, Hong JY, Sunwoo M-K, Sohn YH, Lee PH. Neuroanatomical substrates of visual hallucinations in patients with non-demented Parkinson's disease. *Journal of Neurology, Neurosurgery & Psychiatry* 2012; **83**(12): 1155-61.

11. Watanabe H, Senda J, Kato S, et al. Cortical and subcortical brain atrophy in Parkinson's disease with visual hallucination. *Movement Disorders* 2013; **28**(12): 1732-6.

12. Lee WW, Yoon EJ, Lee JY, Park SW, Kim YK. Visual Hallucination and Pattern of Brain Degeneration in Parkinsons Disease. *Neurodegener Dis* 2017; **17**(2-3): 63-72.

13. Hawrylycz MJ, Lein ES, Guillozet-Bongaarts AL, et al. An anatomically comprehensive atlas of the adult human brain transcriptome. *Nature* 2012; **489**(7416): 391-9.

14. Arnatkevic̆iūtė A, Fulcher BD, Fornito A. A practical guide to linking brain-wide gene expression and neuroimaging data. *NeuroImage* 2019; **189**: 353-67.

15. Markello RD, Arnatkeviciute A, Poline J-B, Fulcher BD, Fornito A, Misic B. Standardizing workflows in imaging transcriptomics with the abagen toolbox. *elife* 2021; **10**: e72129.

16. Desikan RS, Ségonne F, Fischl B, et al. An automated labeling system for subdividing the human cerebral cortex on MRI scans into gyral based regions of interest. *Neuroimage* 2006; **31**(3): 968-80.

17. Fischl B, Salat DH, Busa E, et al. Whole brain segmentation: automated labeling of neuroanatomical structures in the human brain. *Neuron* 2002; **33**(3): 341-55.

18. Shine JM, Halliday GM, Gilat M, et al. The role of dysfunctional attentional control networks in visual misperceptions in Parkinson's disease. *Hum Brain Mapp* 2014; **35**(5): 2206-19.

19. Ignatavicius A, Matar E, Lewis SJG. Visual hallucinations in Parkinson's disease: spotlight on central cholinergic dysfunction. *Brain* 2025; **148**(2): 376-93.

20. Castillo PE, Younts TJ, Chavez AE, Hashimotodani Y. Endocannabinoid signaling and synaptic function. *Neuron* 2012; **76**(1): 70-81.

21. Soria-Gomez E, Busquets-Garcia A, Hu F, et al. Habenular CB1 Receptors Control the Expression of Aversive Memories. *Neuron* 2015; **88**(2): 306-13.
